# Influence of R=Y, Gd, Sm on Crystallization and Sodium Ion Conductivity of Na_5_RSi_4_O_12_ Phase

**DOI:** 10.3390/ma15031104

**Published:** 2022-01-30

**Authors:** Jochen Schilm, Rafael Anton, Dörte Wagner, Juliane Huettl, Mihails Kusnezoff, Mathias Herrmann, Hong Ki Kim, Chang Woo Lee

**Affiliations:** 1 Fraunhofer Institute for Ceramic Technology and Systems Fraunhofer IKTS, Winterbergstrasse 28, 01277 Dresden, Germany; rafael.anton@ikts.fraunhofer.de (R.A.); doerte.wagner@ikts.fraunhofer.de (D.W.); Juliane.huettl@ikts.fraunhofer.de (J.H.); mihails.kusnezoff@ikts.fraunhofer.de (M.K.); Mathias.Herrmann@ikts.fraunhofer.de (M.H.); 2 Department of Chemical Engineering (Integrated Engineering) & Center for the SMART Energy Platform, College of Engineering, Kyung Hee University, 1732 Deogyeong-daero, Giheung, Yongin 17104, Gyeonggi, Korea; hkkim95@khu.ac.kr

**Keywords:** sodium solid state electrolyte, ionic conductivity, sodium solid state battery, glass ceramics processing, impedance spectroscopy

## Abstract

New sodium-based battery concepts require solid electrolytes as ion conducting separators. Besides NaSICON and β-Al_2_O_3_ in the Na_2_O-R_2_O_3_-SiO_2_ system (R = rare earth), a rarely noticed glass-ceramic solid electrolyte with the composition Na_5_RSi_4_O_12_ (N5-type) exists. The present study addresses the investigation of the ionic conductivity of Na_5_RSi_4_O_12_ solid electrolytes sintered from pre-crystallized glass-ceramic powders. The sintering behavior (optical dilatometry), the microstructure (SEM/EDX), and phase composition (XRD), as well as electrochemical properties (impedance spectroscopy), were investigated. To evaluate the effect of the ionic radii, Y, Sm and Gd rare elements were chosen. All compositions were successfully synthesized to fully densified compacts having the corresponding conducting N5-type phase as the main component. The densification behavior was in agreement with the melting point, which decreased with increasing ionic radii and specific cell volume. Alternatively, the ionic conductivities of N5-phases decreased from Y to Gd and Sm containing samples. The highest ionic conductivity of 1.82 × 10^−3^ S cm^−1^ at 20 °C was obtained for Na_5_YSi_4_O_12_ composition. The impact of grain boundaries and bulk conductivity on measured values is discussed. A powder-based synthesis method of this glass-ceramic solid electrolyte using different rare earth elements opens possibilities for optimizing ionic conductivity and scalable technological processing by tape casting.

## 1. Introduction

Since renewables are fluctuating energy sources, stationary energy storage media in the form of batteries and electrolyzers are necessary to enable grid stability and boost the total efficiency of renewable energy utilization. Especially lithium-ion batteries (LIB) with high specific energy densities (100–300 Wh/kg), acceptable cycling life, and high charge/discharge rates are suitable for use in electromobility. Due to the limited occurrence of lithium resources and the steadily increasing price of lithium, supplementary systems with comparable performance must be found [1]. The most promising alternatives is sodium-based electrochemistry, which has lower energy densities, but still shows sufficient performance for applications in stationary energy storage. Furthermore, sodium is advantageous due to its high abundance in the earth’s crust and low price [1,2,3]. The most advanced Na-ion batteries are the Na-S and the Na-NiCl_2_ (ZEBRA). Both cells operate at high temperatures of about 300 °C to ensure that the electrode materials are in a molten state [4,5]. However, the progress of ambient temperature sodium-ion batteries (SIBs) utilizing liquid electrolytes is hindered by dendrite formation, which is more severe in comparison to lithium electrochemistry [6]. For this reason, the focus of current research regarding sodium ion batteries is increasingly concentrated on dendrite-resistant solid state batteries (SSB), which have high energy density and can be used at ambient temperatures [7].

Similar to the high-temperature batteries, solid-state batteries also require a dense, ion-conducting solid electrolyte membrane for the reliable and functional separation of the anode and cathode. This can either be designed as a self-supporting component or as a coating on one of the electrodes. Sintered ceramic solid electrolytes based on oxides (Na-β’’-Al_2_O_3_) or phosphates (Na_1+x_Zr_2_(SiO_4_)_x_(PO_4_)_3-x_—NASICON) are considered suitable to suppress dendrite growth and exhibit high electrochemical stability [8]. The Na-β’’-Al_2_O_3_ solid electrolyte has been used for decades in high-temperature sodium batteries. Na_2_O × 5 Al_2_O_3_, the so-called β’’-aluminate, was invented in 1962 by Thèry et al. [9]. Regarding the review of Fergus at room temperature, an ionic conductivity σ starting ranging between 0.017 and 0.05 S/cm can be expected for a single-crystalline β/β’’ aluminate and polycrystalline β/β’’ aluminate values between 3 × 10^−4^ and 3 × 10^−3^ S/cm [10]. Sintering temperatures for components such as thin membranes or tubes higher than 1600 °C are necessary to produce a dense ceramic [11]. The high sintering temperature is the main drawback of its manufacturing, causing sodium loss and ageing of ceramic furnace linings as well as setter materials.

In 1976, Hong et al. discovered the composition, Na_1+x_Zr_2_(SiO_4_)_x_(PO_4_)_3-x_ (NZSP), which had an ionic conductivity of 0.2 S/cm at 300 °C. By substituting different elements in this composition without changing the crystal structure, higher ionic conductivities for many compositions could be realized. These materials are grouped under the term “NASICON,” which stands for “Na superionic conductor.” The ionic conductivity of this class of solid electrolytes varies between 1 × 10^−4^ and 1 × 10^−3^ S/cm at 25 °C [12]. Although numerous compositions and stoichiometric variations of the basic NASICON has been synthesized and characterized, their sintering process remains challenging, and several attempts have been investigated to reduce the sintering temperatures by using field-assisted sintering (FAST), a cold sintering Process (CSP), or the additions of sintering additives for liquid phase sintering (LPS) [11,13,14]. For conventional sintering processes, temperatures above 1200 °C (up to 1300 °C) together with several hours of soaking time are necessary to achieve dense microstructures. Such conditions lead to evaporation of Sodium and Phosphor from the specimens, resulting in changed compositions, unwanted phases, and reduced conductivities [15].

One attempt to reduce the sintering temperature of NASICON has been undertaken by using a glass processing route followed by the crystallization process. Susman et al. reported a so-called NASIGLAS material with reduced ZrO_2_ content and a modified composition Na_1+x_Zr_2-x/3_Si_x_P_3-x_O_12−2x/3_ with x = 3, which can be manufactured as glass and has a conductivity of approximately 1.5 × 10^−3^ S cm^−1^ at 300 °C and 1.3 × 10^−7^ S cm^−1^ at 25 °C [16]. Glasses with lower x-values (x = 2.25–3) synthesized by Niyompan et al. showed similar conductivities at 300 °C and also a tendency for crystallization under thermal treatment [17]. However, the high content of crystalline, highly conductive NASICON phase by a subsequent crystallization step of NASIGLAS could not be demonstrated up to date.

In 1969, Maksimov et al. discovered the structure Na_5_RSi_4_O_12_ (R = La-Lu and Y) with a rhombohedral crystal structure (R3c^−^) [18]. Shannon et al. [19] performed a systematical study on the conductivity of hydrothermally sintered Na_5_RSi_4_O_12_ compositions for R = Er, Y, Ho, Dy, Gd, Eu, and Sm and found an increasing ionic conductivity for increasing the radii of trivalent cations from Sc^3+^ to Sm^3+^ ranging from 3 × 10^−3^ S/cm (Sc^3+^) to 0.1 S/cm (Sm^3+^) at 200 °C. Beyeler et al. [20] performed room temperature conductivity measurements on the single crystal Na_5_YSi_4_O_12_ and obtained an ionic conductivity parallel to the c axis of 3.2 × 10^−3^ S/cm. Perpendicular to the c axis, even higher conductivity values of 8 × 10^−3^ S/cm were measured. However, an ionic conductivity of 1 × 10^−3^ S/cm was obtained for the Na_5_RSi_4_O_12_ phase in polycrystalline microstructures at room temperature [20]. The crystallization of the Na_5_RSi_4_O_12_ phase from glasses with composition Na_3.2_R_0.9_Si_2.1_P_0.1_O_8.7_ with R=Er, Y, Gd, and Sm has been investigated by Banks et al. [21] and led to the formation of sodium conducting glass-ceramics. The possibility of synthesizing inorganic solid electrolytes as glass-ceramics, which have conductivities comparable to NASICON and Na-β’-Al_2_O_3_, provides an interesting alternative to these established solid electrolytes and their complex manufacturing processes.

Since 1985, Yamashita’s group has worked on manufacturing and optimizing the properties of Na_5_YSi_4_O_12_-based electrolytes [22]. By tuning Na_3+3x−1_Y_1-x_P_y_Si_3-y_O_9_ composition, it was possible to increase the volume content of the conductive N5 phase [23]. In 2012 Okura et al. [24] demonstrated the predominant crystallization of the N5 phase from quenched glass samples with proper Na_3+3x-y_Y_1-x_P_y_Si_3-y_O_9_ (NAPSY) stoichiometry. However, the presence of additional non-conducting phases such as N8 (Na_8__.__1_YSi_6_O_18_), N3 (Na_3_YSi_2_O_7_), and N9 (Na_9_YSi_6_O_18_) have been observed. Despite the crystallization ability of the high content of the N5 phase, the presence of the isolating N3 and N9 phases press the value of the total conductivity to 0.066 S cm^−1^ at 300 °C [24]. These phases, as well as a residual glass phase at grain boundaries, have ionic conductivity 1 to 3 orders of magnitude lower than the N5 target phase [25]. A recently published review article by Okura and Yamashita [26] provides a good guideline for R&D activities on NAPSY based sodium-ion superconductors produced by recrystallization from the glass phase. Similar to the β-aluminates and NASICON, oxide glass-ceramics are also resistant to high temperatures and sustain contact with metallic sodium [27]. According to Danzer, the mechanics of this material’s sintered powder compacts have been investigated by the 3-Ball-on-ball method, and a bending strength up to 60 MPa was determined [28].

Previously, Wagner at al. demonstrated the possibility of manufacturing thin tape casted substrates from NAPSY glass-ceramics with a parent composition corresponding to the N5-type phase [29]. It was found that the crystallization of P-containing N5 phase in NAPSY led to highly ionic conducting grains and the substantial enhancement of total ionic conductivity even in the presence of isolating secondary phases [30]. According to the early work of Shannon et al. [19], Sm and Gd provide the option for enhancing the ionic conductivity of electrolytes with high N5 phase content. The purpose of this work is to elicit the potential of ionic conductivity enhancement at ambient conditions in sintered glass-ceramic microstructures of the stoichiometric N5-composition with rare earth cations with larger radii in comparison to Y^3+^ (Gd^3+^ and Sm^3+^).

## 2. Materials and Methods

Na_2_CO_3_ (AnalaR NORMAPUR, VWR), SiO_2_ (Millisil W8, Quarzwerke Frechen, Frechen, Germany), and rare earth metal oxides Y_2_O_3_ (Grade C, H.C. Starck, Goslar, Germany), Gd_2_O_3_ (Alfa Aesar, Kandel, Germany), and Sm_2_O_3_ (Alfa Aesar, Kandel, Germany) have been used as raw materials to synthesize amorphous glass with composition corresponding to Na_5_RSi_2_O_12_ stoichiometry. The powders with molar ratio Na_2_CO_3_:SiO_2_:R_2_O_3_ = 35.7:7.2:57.1 were mixed with a dry tumble mixer (TURBULA, Willy A. Bachofen AG, Muttenz, Switzerland) for 30 min. The well-mixed powders were melted in a platinum crucible at 1350 °C and quenched on a brass block to room temperature. The resulting glass frit was transparent and colorless for yttria and gadolinia and had a yellowish color for the samaria sample. The obtained glass samples were milled down and crystallized to the N5 phase (N5-Y for Na_5_YSi_4_O_12_, Na_5_GdSi_4_O_12_ for N5-Gd, and Na_5_SmSi_4_O_12_ for N5-Sm) using corresponding heat treatments summarized in Table 1. The temperatures for the pre-crystallization of N5-Sm and N5-Gd glasses were defined based on preliminary caloric analyses carried out on the milled powders. Accordingly, annealing temperatures were selected that exceeded the crystallization temperature of the glasses but stayed at least 100 K below their corresponding melting point. The corresponding analysis of crystallization behavior of N5-Y glass and the definition of favorable N5 phase crystallization conditions are described in our previous studies on this material [28].

After crystallization, the frit was crushed in a mortar and milled for 1 h in ethanol using an attritor (NETZSCH-Feinmahltechnik GmbH, Selb, Germany). The particle size distributions were determined by laser diffraction in a range between 20 nm and 2000 µm (Mastersizer 2000, Malvern Instruments Ltd., Malvern, UK). The characteristic particle sizes of the glass powders after milling were for d_10_ = 0.6–0.7 µm, d_50_ = 1.6–1.8 µm, and d_90_ = 3.7 µm for N5-Y and N5-Gd and d_90_ = 4.6 µm for N5-Sm. The powders were dried and pelletized with 25.5 MPa to discs with a thickness of 1–2 mm and a diameter of 20 mm. The green density of pellets was measured from their weight and discs dimensions. The discs were sintered in air on platinum foils under an ambient atmosphere with a heating rate of 5 K/min. The sintering conditions for different compositions are summarized in Table 2. They represent the results of a series of tests conducted with the aim of achieving the highest possible densities in the microstructures.

The shrinkage of the powder has been analyzed by optical dilatometry using a hot stage microscope (Hesse Instruments, Osterode am Harz, Germany) which analyzes the changes of the projected and photographed shadows of the sample according to DIN 51730. This method is well described by Pascual and Durán [31].

Samples for analysis of the ionic conductivity (by electrochemical impedance spectroscopy—EIS) were sputtered with gold (acting as ion blocking electrode), placed in ECC Std test cells (EL-CELL GmbH, Hamburg, Germany), and put in the temperature chamber (Binder GmbH, Tuttlingen, Germany). Impedance measurements were carried out by a potentiostat VMP3 (BioLogic Science Instruments, Seyssinet-Pariset, France) with an amplitude of ac perturbating voltage signal of 25 mV in a frequency range between 100 mHz and 1 MHz. Impedance spectra were recorded in a temperature range from −10 to 30 °C in 10 K steps. The measurement program used was EC Lab V11.36. Prior to each measurement, the cell was tempered for 1.5 h to reach the appropriate temperature inside. The impedance spectra were evaluated using RelaxIS Software vers. 3.0.17.10 from RHD GmbH (Wedemark, Germany).

Density measurements of the sintered pellets were performed according to the Archimedean principle in ethanol with a Sartorius CP2245 balance and a density determination kit. The theoretical density of the fully crystallized material was measured from milled samples by helium pycnometry.

For the quantitative XRD analysis, powder compacts were heat-treated under the same conditions as those used for impedance measurements. Afterwards, the samples were ground down to a particle size < 63 µm and mixed with 33.333 wt.% pure Si powder as internal reference for calculation of the amorphous phase content. The diffractograms were recorded by a X-ray-diffractometer (D8 Advance, Bruker AXS) with Cu Kα-radiation and a LynxEye position sensitive detector (PSD). The samples were measured in a range of 10–90° (2θ, with a step width of 0.03° and a measuring time of 3 s (PSD). The qualitative analysis was performed with the software DIFFRAC.EVA (Version 5.2., Ltd., Bruker AXS, Karlsruhe, Germany) with an ICDD PDF(2021)-database. The quantitative analysis was performed using the Topas 6.0 software package. For the quantitative analysis of Na_5_YSi_4_O_12,_ the structural data set [32] was used. In the case of Na_5_SmSi_4_O_12_ and Na_5_GdSi_4_O_12,_ the Y data were used with an adjustment of the lattice parameters. The same procedure was used for the cubic Na_16_(Na_0.5_Y_0.5_)Y_2_Si_12_O_36_ phase, which was described by Többens et al. [33]. The uncertainty of the values determined for the crystalline phases is limited to ca. 1 wt.% and for the residual amorphous phase to 3–5 wt.%.

Microstructural analysis was performed with polished cross-sectioned samples using a field emission scanning electron microscope (FE-SEM, NVision 40; Carl Zeiss SMT, Oberkochen, Germany). For the preparation of the samples, a combination of mechanical grinding steps and final ion-polishing by argon-ions was used (RS 101, Bal-Tec, Balzers, Liechtenstein). The micrographs shown in this paper were recorded in the atomic-number-specific back-scattered electron (BSE) contrast. The FE-SEM is equipped with an energy dispersive X-ray analysis system (Inca x-sight; Oxford Instruments, Abingdon, UK), which allows a quantitative detection of elements.

## 3. Results

### 3.1. Shrinkage Behavior of Crystallized Powders

**Figure 1:**
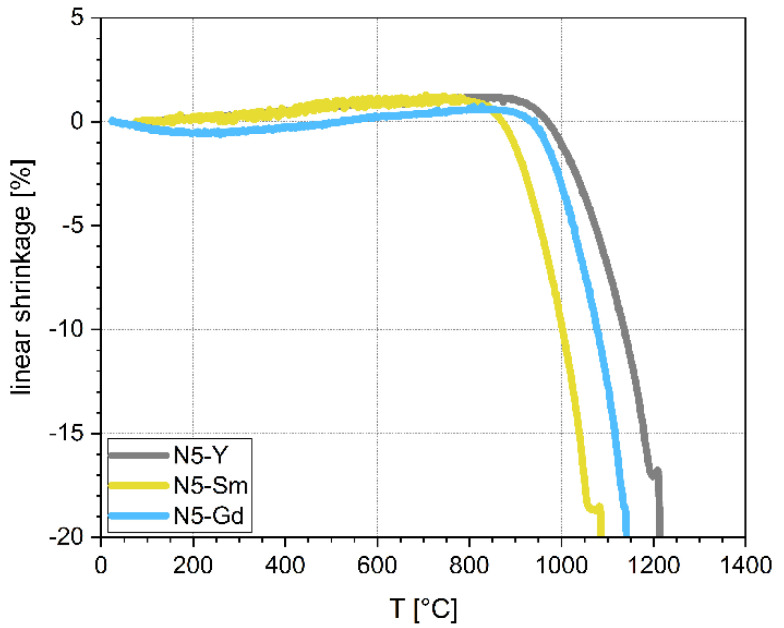
Shrinkage behavior of pre-crystallized powders.

The results of the shrinkage investigations of the pre-crystallized powders are shown in Figure 1. All samples show expansion up to 800 °C, which relates to the thermal expansion of the corresponding material and minor foaming effects due to binder burn out. The samples sinter within a temperature range between shrinkage start and melting point (temperature window of 200 K to 250 K), determined by the characteristic temperatures summarized in Table 3. The characteristic temperatures “shrinkage start” and “shrinkage end” are determined by the analysis of the shrinkage curve using the software of the Hesse Instruments hot stage microscope. It should be noted that the temperatures of shrinkage end and the occurrence of the first melting phase come very close to each other. As a result, we had to choose the maximum possible sintering temperatures for the samples just below the melting point to avoid any melting or decomposition of the conductive N5-type phases. The melting temperature decreased with increasing cation radius resulting in the lower sintering temperature of corresponding powders, meaning that larger cations weaken the covalent bonds in the material.

### 3.2. Density and Porosity

The pycnometric density of the pre-crystallized powders is summarized in Table 4. Compared to the crystallographic densities calculated from ICSD and Pearson databases, the experimental data show only a slight systematic deviation to lower values. The lattice parameters of the rhombohedral N5 phase increase from Y^3+^ to Sm^3+,^ showing a weakening of covalent bonds and a widening of the lattice, which agrees with the observed decrease of melting temperature.

**Figure 2:**
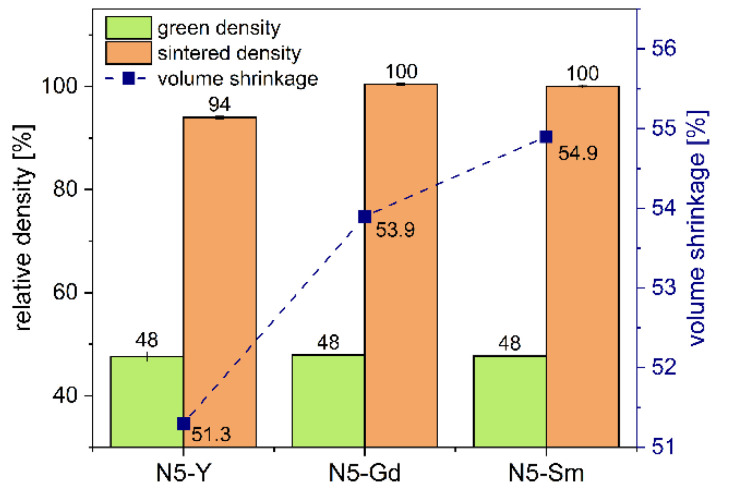
Green density, shrinkage and sintered density of powder compacts.

The green density values of the manufactured pellets as well as the sintered density and shrinkage are summarized in Figure 2. The residual porosity was calculated from the pycnometric density of the powder used and the sintered density of the pellet according to Equation (1) giving the total (open and closed) porosity of the sample. By using these data, it is possible to calculate the open porosity from the sintered samples without any need for knowledge of the phase composition. The uncertainty of the results received by the Archimedes method is 0.2% and <1% for data measured by helium pycnometry.

(1) $$Porosity=100\%-\rho_{rel}=100\%-\frac{\rho_{sint}}{\rho_{pycno}}\cdot 100\%$$

The open porosity has been determined during the measurement of Archimedean density and is zero for all investigated samples. For the sample N5-Y, a value of 6% of closed porosity was determined. N5-Gd and N5-Sm samples had a porosity below the uncertainty limit of the performed calculations (<1%).

The absolute value of closed porosity decreased from N5-Y (6 vol.%) to zero for N5-Gd and N5-Sm composition in agreement with the trend observed for the melting point of their corresponding compositions.

### 3.3. Phase Analysis

**Figure 3:**
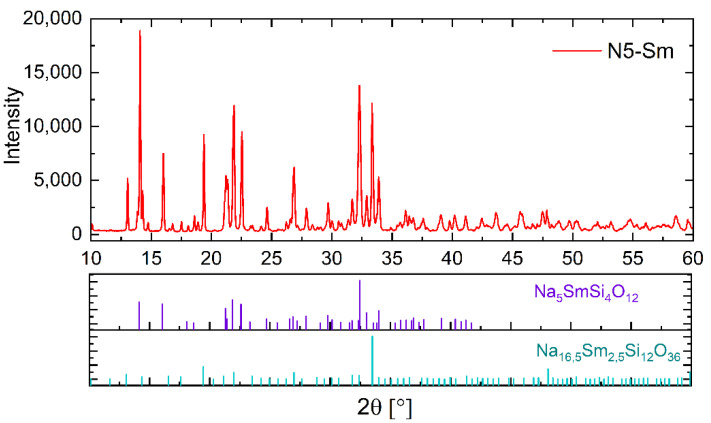
XRD pattern of sintered N5-Sm sample in comparison with PDF cards of the N5 and N16.5 phases.

The determination of the crystalline phases in the pre-crystallized powders and the sintered samples was made by analysis of XRD diffractograms. For example, we only present in Figure 3 the XRD pattern of the Sm-substituted sintered samples together with PDF cards of Na_5_SmSi_4_O_12_ and Na_16.5_Sm_2.5_Si_12_O_36_. The main phase detected in all NaRSiO XRD patterns is the targeted N5 phase (Na_5_RSi_4_O_12_ (R=Y, Gd, Sm)) with a rhombohedral lattice structure. Additionally, a minor amount of a cubic Na_16.5_R_2.5_Si_12_O_36_ phase (N16.5) could be determined by Rietveld refinement. The determination of this minor phase is based on the results of Többens et al. [33]. They tried to synthesize the formerly mentioned N9 phase through crystallization of an Yttrium substituted, stoichiometric parental glass composition. The incomplete crystallization led to the formation of a cubic structure having the slightly deviating composition Na_16.5_Y_2.5_Si_12_O_36_. From the crystallographic point, this phase could be a solid solution with the N5 phase as the Y-rich boundary composition. Therefore, the cubic phase represents either a cubic high-temperature modification of the N5 phase or a compound slightly richer in sodium (Na_16.5−3x_Y_2.5+x_Si_12_O_36_ (0 < x < 0.5). Both phases, Na_16.5_Y_2.5_Si_12_O_36_ and N5, consist of twelve-membered rings of corner-connected SiO_4_-tetrahedra but with a slightly different ordering of cations. This would have to be considered in more detail in future investigations. For Sm containing pre-crystallized powders, an apatite structure Na_0.5_Sm_4.5_(SiO_4_)_3_ with a fraction of 0.8 ± 0.2 wt.% has been determined by Rietveld refinement. This determined content exhibits a high error due to the peak overlaps with the main N5 phase. In sintered samples, no peaks from the apatite phase have been detected. Other sodium rare earth silicate phases, such as Na_3_RSi_2_O_7_ and Na_9_RSi_6_O_18_ reported by Okura et al. [34] and Wagner et al. [28], could not be detected in pre-crystallized powders or in sintered samples. The results of the quantitative analysis of crystalline phase content in the sintered samples are summarized in Table 5. The deviations given are the statistical uncertainty of determining the crystalline phase content. The methodological uncertainties due to deviation in the composition and structure of Na_16_(Na_0.5_R_0.5_)R_2_Si_12_O_36_ could be in the range of 1 wt.%. According to Figure 3, we added graphical presentations of the results of XRD-analysis of the compositions N5-Y and N5-Gd as Supplemental Information Figures S1 and S2.

Regarding the quantitative determination, it should be noted that the values do not have to correspond exactly to the phase content present in the sample since the calculated mass fraction depends on the sodium ion occupation of corresponding lattice sites within singular crystalline phases. Since these known structures in this system have both local and freely mobile sodium ions, an uncertainty in the possible compositions exists, which results in small deviations from the calculated content. Nevertheless, the obtained indicative results can be used for comparative evaluation of the weight fraction of a certain phase in investigated samples. Furthermore, in a few diffractograms, one or two peaks or peak shoulders could not be assigned to any PDF cards and resulted in some uncertainty estimated to be less than 1%. These could correspond to the rare-earth-rich inclusions found in the micrographs (compare Section 3.4). Clearly, the amount of N16.5 phase increases from ca. 4 wt.% in the N5-Y sample to ca. 34 wt.% in the N5-Sm pellet.

### 3.4. Microstucture Analysis

**Figure 4:**
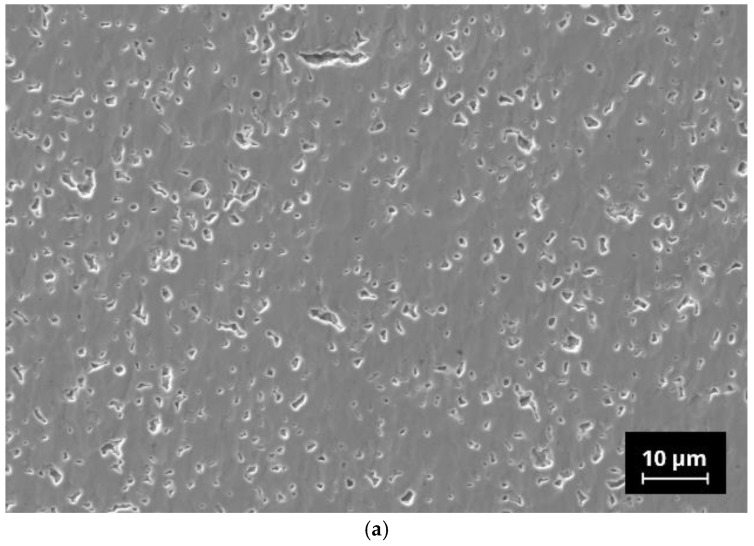

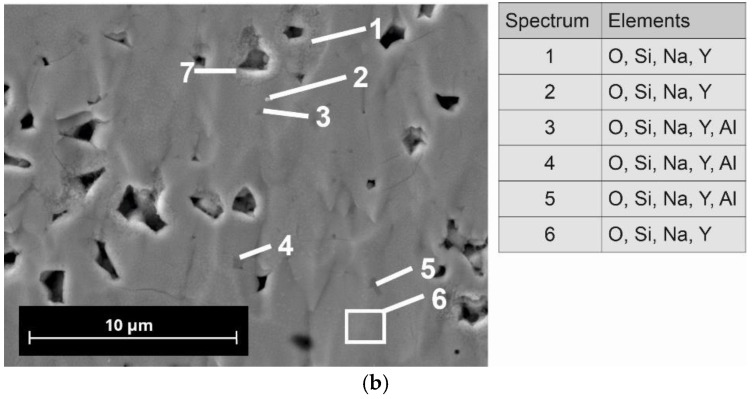
Microstructure of N5-Y pellet sintered at 1120 °C/2 h: (**a**) micrograph of polished cross section; (**b**) BSE image with indicative EDX analysis results.

Micrographs of sintered N5-Y sample show small (<5 µm) closed pores with irregular shapes in the microstructure, which are homogeneously distributed in the bulk of the sample (Figure 4). At a higher magnification, some dark grey areas can be seen in the back scattering electron (BSE) mode imaging. EDX analysis of the selected points shows the presence of Al in these areas. Al was not intentionally added to the raw powders and probably originated from abrasion during attrition milling. Other EDX spectra correspond mainly to the N5-Y phase, however no quantitative analysis of EDX data has been performed.

**Figure 5:**
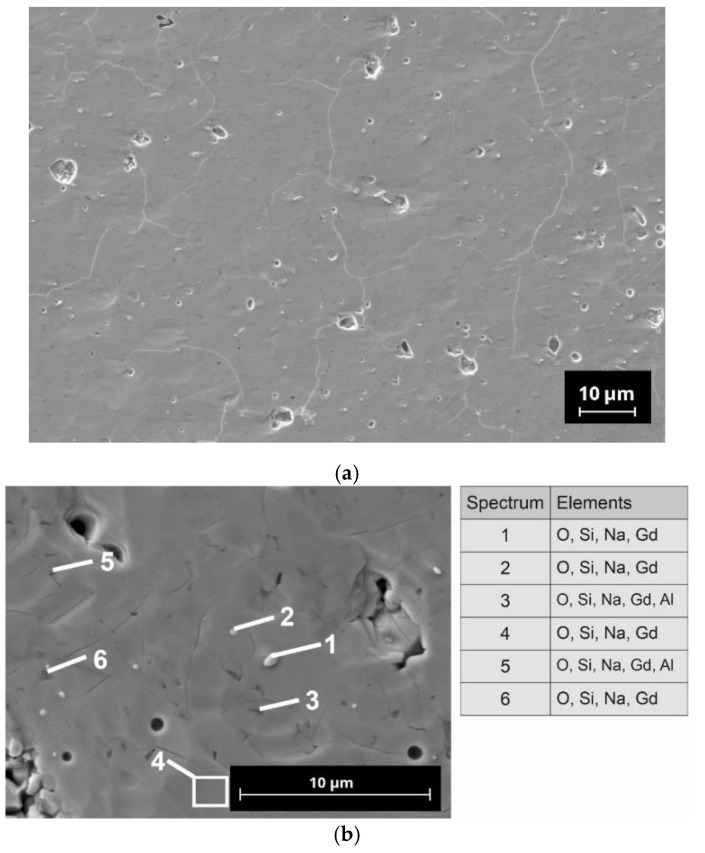
Microstructure of N5-Gd pellet sintered at 1120 °C/0.5 h: (**a**) micrograph of polished cross section; (**b**) BSE image with indicative EDX analysis results.

The microstructure of the N5-Gd sample, shown in Figure 5, has a considerably lower amount of pores in comparison to N5-Y. This observation correlates well with the porosity values determined for both samples. In this sample, Al containing phases have been detected. Additionally, small single-light inclusions were detected in the microstructure. It is assumed that these phases have higher Gd content, which causes the contrast in the imaging.

The microstructure of the N5-Sm sample is like that of N5-Gd. In both cases, the number of light phases in the observed cross-section of this sample is considerably increased, which indicates the presence of some phases with higher Sm and Gd contents. Interestingly, the rare earth element-rich phases correlate with the N16.5 phase content in the samples N5-Sm and N5-Gd determined by Rietveld refinement.

### 3.5. Ionic Conductivity

The ionic conductivity of the sintered pellets was determined from analysis of impedance spectra. The main target of this analysis is to determine the grain and grain boundary resistivity, which are present in the spectra and physically described by the brick-layer model [35]. Due to possible overlap of impedance responses resulting from grain boundaries and electrode contributions [30], the main focus of this work is directed toward the separation of the contribution of bulk conductivity of the N5 phase and the estimation of its value for ambient temperature as well as the activation energy for N5-Y, N5-Gd, and N5-Sm pellets. Additionally, based on results published in the literature so far and our results, we cannot estimate the exclusive ion conducting properties of the the N9 or N16.5 phase as no information has been found which makes direct statements on the ionic conductivity of this phase. Hence, for this study, we rate this phase as a non-conducting constituent in the sintered microstructures.

#### 3.5.1. Spectra Analysis

**Figure 6:**
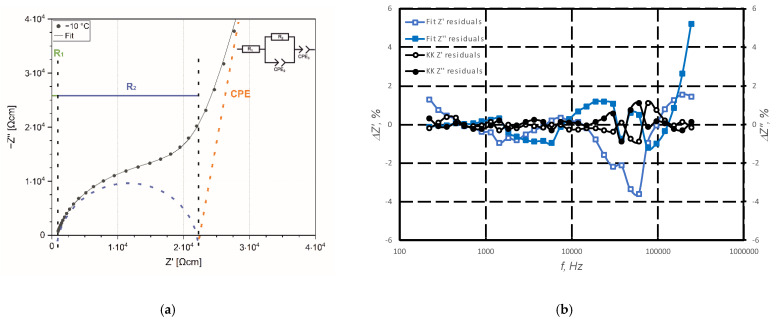
Analysis of EIS for N5-Y sample: measured data at −10 °C and fitted spectra (**a**) and relative deviations between measured values, Kramers-Kronig (KK) test and fitted values (**b**).

The impedance spectra of the Na5-Y pellet at −10 °C is shown in Figure 6a. In general, following main contributions to the spectrum were found:

- Bulk resistivity of grains with relaxation frequency of 100 MHz [36];
- Grain boundary resistivity with relaxation frequency of 1 kHz to 10 kHz;
- Electrode interface contribution with relaxation frequency 100 Hz to 0.1 Hz;
- Blocking electrode contribution at frequencies < 1 Hz.

In order to extract numerical values for the contributions of the grains and the grain boundaries to the ionic conductivity from the impedance spectra, it is necessary to fit them using appropriate equivalent circuits. To account for the distribution of relaxation time constants arising from the inhomogeneity of microstructure and material properties like dielectric permittivity and conductivity, several (R-CPE) sub-circuits, connected in series, are used to describe the suppressed grain and grain boundary semi-circles instead of (R-C) elements. The suppression degree (*α*) in (R-CPE) circuit is a measure of homogeneity for relaxation time constants (highest homogeneity at *α* = 1) resulting from materials properties like dielectric permittivity and conductivity [37]. The detailed analysis of the separation of those contributions is given in [30] even if the contribution of grain boundaries and electrode interface has not been clarified completely. Since the response of the N5 phase in the impedance spectra is seen at ca. 10–20 MHz, the (R-CPE) element corresponding to this arc can be substituted with an ohmic resistance assuming the impact of grain conductivity. As fully crystallized samples have been used, the blocking electrode behavior can be described with a CPE with an expected slope of >65° [30]. The indicative analysis of the impedance spectra was made by a simplified equivalent circuit consisting of serial connection of resistance (R-CPE) and constant phase element (CPE). Figure 6b shows the residuals between measured spectra and Kramers–Kronig transformation as well as the fitted spectra.

#### 3.5.2. Fit Results and Their Interpretation

**Figure 7:**
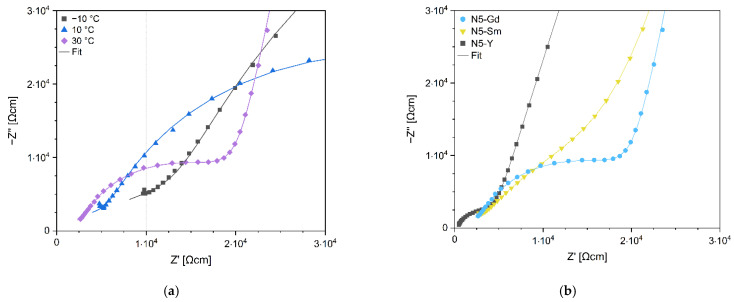
Nyquist plots for impedance temperature dependence for sample N5-Gd (**a**) and impedance dependence on composition of Na_5_RSi_2_O_12_ phase for R=Y, Gd and Sm at 30 °C (**b**).

The temperature dependence of the impedance spectra of N5-Y is shown in Figure 7a and the comparison of impedance spectra of N5-Y, N5-Gd and N5-Sm samples at 20 °C in Figure 7b. The values of specific parameters obtained from fitting the impedance spectra of the N5-Sm sample at different temperatures normalized to the sample thickness and electrode area are summarized in Table 6. The conductivity values obtained from high-frequency impedance were assumed to correspond to the contribution of the ionically conductive N5 phase.

The low frequency contribution cannot be clearly assigned to grain boundary only, and the physical origin of this arc should be clarified in further research. To get more insight into the origin of this arc, the relaxation frequency and apparent capacitance have been calculated from following equations [38]:

(2) $$f_{0,LF}={\left(2\pi R_{LF}C_{ap}\right)}^{-1}={\left(2\pi \sqrt[{\alpha }]{R_{LF}\cdot Q_{LF}}\right)}^{-1}$$

(3) $$C_{ap}=\sqrt[{\alpha }]{R_{LF}\cdot Q_{LF}}/R_{LF}$$

The high capacitance values obtained (see Table 6) from fit results and its temperature dependence (the dielectric constant should be independent of temperature in such a narrow frequency range) do not allow for the clear identification of this contribution as grain boundary only. This impact clearly correlates with the number of secondary phases present in the sample. The detrimental influence of Al impurities detected by EDX also cannot be excluded.

The activation energy for the low frequency arc can be calculated from the temperature dependence of the obtained parameters. The activation energy of the resistivity resulting from low frequency is in the range of ca. 0.3 eV for all samples. For further interpretation of measured data, the high frequency contribution assigned to the impact of the N5 phase will be analyzed in detail.

#### 3.5.3. Ionic Conductivity of N5 Phase

The conductivity of the N5 phase can be calculated from fitted normalized values using its volume content (*x*_N5_) and tortuosity factor (*τ*_N5_) describing the elongation of diffusion path through the sample:

(4) $$\sigma_{N5}=\tau_{N5}{\left(x_{N5}R_{G}\right)}^{-1}$$

Using the values for the content of crystalline phases in wt.%, their crystallographic densities (i.e., 2.87 g cm^−3^ for N5-Y and 2.91 g cm^−3^ for N16.5-Y) from Table 5 and porosity of the corresponding samples (see Figure 2) the volume content of all constituents can be calculated. The results of these calculations are summarized in Table 7. Since all samples have high densities and N5 phase contents, the tortuosity of $\tau_{N5}$ = 1 has been assumed for our estimations. The detailed analysis of tortuosity for N5-Y, N5-Gd, and N5-Sm samples has not been performed. It is possible to determine tortuosity by 3D microstructure reconstruction; such analysis can be a topic of separate investigation. Some reference values could be considered to evaluate the influence of this factor on calculated conductivity values. The tortuosity values for the two-phase microstructures of La_0,6_Sr_0,4_Co_0,8_Fe_0,2_O_3-x_ analyzed by 3D reconstruction resulted in a tortuosity of the electronically conducting phase of 1.1 for 7% porosity [39], 1.23 for 24.9% porosity [40], and 1.48 for 31.16% porosity [41]. Since in this study we focus on the conductivities of the respective N5 phases and assess the N16.5 phase as non-conductive according to the current state of knowledge, we assume, as a first approximation, a 2-phase structure, of which only the N5-phase contributes to conductivity. Thus, for the estimation of the tortuosity of these microstructures, we can refer to the values of the previously mentioned publications. However, the tortuosity factors also depend on grain size and distribution of microstructure constituents and cannot be transferred from the reported microstructure to another one. The reported grain size of La_0,6_Sr_0,4_Co_0,8_Fe_0,2_O_3-x_ was 1.55 µm for the sample with 24.9% porosity and 1.3 µm for the sample with 31.16% porosity, which is much lower in comparison with the grain size of the N5 phase in our samples (see Figure 5a). The true tortuosity factor corrected conductivity value for N5-Gd and N5-Sm samples will be higher than the calculated values with our simplified assumption of $\tau_{N5}$ = 1.

**Figure 8:**
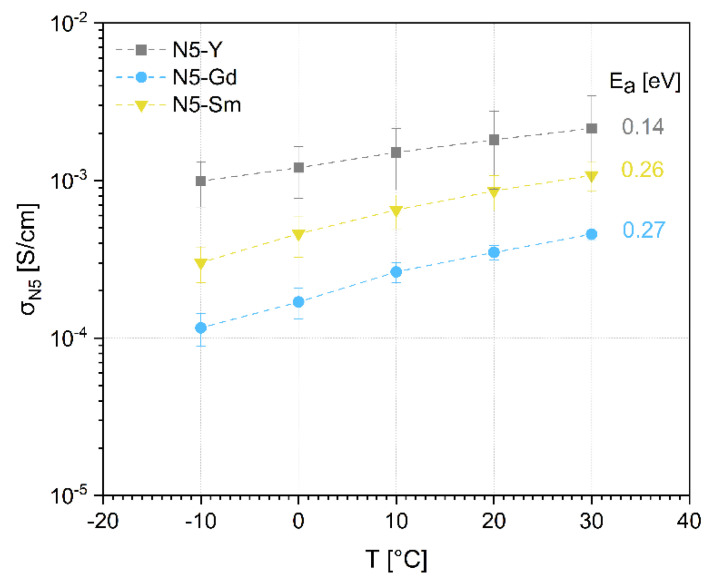
Temperature dependence of ionic conductivity of N5 phase for N5-Y, N5-Gd and N5-Sm.

The temperature dependence of ionic conductivity of N5 phase for different compositions is shown in Figure 8.

The activation energies calculated from (*σ*_Ν5_*T*) vs. 1/*T* plots range from 0.14 eV for N5-Y phase to 0.27 eV for N5-Gd phase. The values have been calculated by using a linear regression of the individual data points according to the Arrhenius equation (Equation (5)):


(5) $$\sigma_{N5}=\frac{\sigma_{0}}{T}\cdot \text{exp}\left(-\frac{E_{\alpha }}{k_{B}T}\right)$$

The absolute value of the ionic conductivity of the N5-Y phase from measurements up to 100 kHz at 20 °C (1.82 mS cm^−1^) is lower than the corresponding conductivity value obtained on phosphor-doped N5-Y composition from high-frequency measurements up to 100 MHz (4 mS cm^−1^) but in better agreement with the total conductivity value of 1.85 mS cm^−1^ including the grain boundary contributions visible at frequencies > 200 kHz [36].

## 4. Discussion

The glass ceramic route has been chosen for the synthesis of the parental compositions of the solid electrolytes. According to our previous studies on the Y-substituted type of this material (N5-Y), it has been found that a pre-crystallization of the parental glass powder prior to the sintering of compact specimens is advantageous in achieving dense microstructures [28]. The sintering of glass-based powder compacts is driven by the viscous flow at temperatures above the glass transition temperature (Tg). In the case of the chosen N5-Y composition, the crystallization occurs within the same temperature windows where the densification takes place and consequently results in only porous microstructures. Preliminary studies with the Sm- and Gd-substituted materials revealed a similar behavior that made it impossible to sinter dense microstructures directly from glass-based powder compacts. From the optical dilatometry it is possible to deduce characteristic temperatures for sintering individual compositions (Table 1). With respect to the ionic radii of the substituted elements Y, Gd, and Sm, the sintering temperature decreases with an increasing ionic radius. As one explanatory approach, this behavior could be attributed to the widening of the crystal lattice of the N5-type phases with increasing ionic radius and an enhanced sintering activity. Also, the temperature windows, in which the densification takes place, become narrower for an increasing ionic radius (N5-Y: Δ*T* = 237 K, N5-Gd: Δ*T* = 215 K; N5-Sm Δ*T* = 192 K). Additionally, we see that the maximum sintering temperature and the melting point lie quite close, especially for N5-Y and N5-Gd. Therefore, the sintering temperatures need to be chosen somewhat below the melting point to avoid any melting and consequently the destruction of the conductive N5-phases. In the case of N5-Y, a soaking time of 2 h at a constant sintering temperature still leaves a residual porosity in the microstructure. In contrast, for N5-Gd a dwell time of 30 min at the same sintering temperature is sufficient to achieve a full densification. According to the lower melting point of N5-Sm, the sintering temperature was also reduced by 70 K and a dwell time of 30 min is enough to densify the powder compacts.

Regarding the formation of the multiphase microstructures, in the case of N5-Gd and N5-Sm, the considerably higher fractions of N16.5 phases compared to N5-Y are worth discussing. On the one hand, this might be attributed to the shorter holding times at the sintering temperature. It is known from the Yttrium substituted system that the non-conducting phases N3 and N9 are formed first at sintering temperatures below 900 °C [28,34]. At higher temperatures the conducting N5-phase crystallizes from the parental glass matrix, and a transformation from the formerly formed non-conducting phases is likely. This behavior has been observed for P_2_O_5_-containing compositions. Additionally, in the case of our ternary composition, N5-Y, after sintering, the microstructure contains only a low amount of the N16.5 phase. If it can be assumed that N16.5 represents the cubic high temperature phase of the N5-phase, it is possible that the sintering conditions chosen for N5-Gd and N5-Sm are suitable to stabilize this phase. Therefore, further experiments must clarify if lower sintering temperatures or additional thermal treatments at lower temperatures can initiate phase transformations in the direction of N5.

One the other hand, the specimens N5-Sm and N5-Gd contain numerous rare-earth-rich precipitations distributed homogenously in the microstructures. Because the respective N5 phases are richer in rare earth ions than the N16.5 phases, it is also conceivable that these rare earth ion-rich precipitates prevent the formation of higher proportions of N5 phases. A reason for the formation of these precipitates cannot be given at this stage. We are aware of the fact, that for N5-Gd and N5-Sm further optimization of the sintering conditions in the direction of longer sintering profiles or adjusted temperatures is necessary to realize higher contents of the N5-phases. However, in view of the holding times of several hours during sintering processes often mentioned in the literature in the case of NaSICON or β-Al_2_O_3_, distinct advantages can be assumed for this class of materials regarding the reduced evaporation of sodium.

When examining the phase composition of the investigated samples, in addition to the conductive N5 phase, another, rarely described N16.5 phase has been detected [33]. In our previous studies and in those of Yamashita and Okura, this phase has not been detected so far [28,30,34]. This may be related to the fact that in the literature, mainly compositions of this material class have been investigated, which deviate from the exact N5 stoichiometry. Yamashita and Okura, who have contributed the predominant part of knowledge on this class of materials, have mainly investigated compositions with nominal stoichiometries of Na_3+3x-y_Y_1-x_Si_3-y_P_y_O_9_ with x = 0.2…0.5 and y = 0…0.45. In addition, our group was focused on a composition having an x-value of 0.25 which corresponds to the stoichiometric N5-type composition with an additional amount of 3 mol% of P_2_O_5_ as an sintering additive [28,30,42]. It is to be noted that N5-Y, with approx. 4 wt.%, contains a significantly lower proportion of the N16.5 phase than the compositions N5-Gd and N5-Sm. As we do not know the thermophysical properties of the N16.5-type phase, it is also possible that the increased amounts of this phase in N5-Gd and N5-Sm are responsible for their increased sintering activity in comparison to N5-Y. We assume that due to the similar composition of the N5 and the N16.5 phases in the present SEM images, no visual differentiation is possible. In the case of N5-Y, the porosity is very homogenously distributed, indicating only a neglecting coarsening effect of the grains. The EDX analysis reveals the presence of aluminum impurities whose origin we must assign to the abrasion of the alumina containers used for ball milling. However, as the same preparation procedure has been applied to all three compositions, we do not assume that the aluminum impurities are responsible for the formation of the high N16.5 contents in case of N5-Gd and N5-Sm. The inhomogeneous distribution of the aluminum indicates the formation of additional phases which cannot be identified by XRD because their content is below the detection limit of this method.

The molar lattice volume increases and the melting point of N5 phase decreases in the series from Y to Sm. This correlates well with the increasing radii of trivalent cations from 0.900 Å for Y^3+^ to 0.938 Å for Gd^3+^ and to 0.958 Å for Sm^3+^ [43]. However, the observed dependence of ionic conductivity for the N5 phase with Y, Gd, and Sm cations, extracted from the impedance measurements up to 100 kHz shows a different trend with decreasing conductivities. The investigations of microstructure of the samples reveal the increasing content of N16.5 phase from Y to Sm in investigated Na_5_RSi_4_O_12_ compositions. There are two possibilities explaining this observation:

1. Composition with Na_5_YSi_4_O_12_ has the highest bulk ionic conductivity and lowest activation energy due to higher Na^+^-ion mobility in the lattice in comparison to other compositions;
2. The measured conductivity at 100 kHz still includes grain boundary contributions, visible at higher frequencies, which are strongly affected by the presence of the N16.5 phase at grain boundaries.

A remaining issue is judging the ion-conducting properties of the individual N16.5-phases. If N16.5 represents a cubic high-temperature phase of the N5-modification it could be possible that its specific ionic conductivity is even higher than that of the N5-modification. If the phase has a special ordering of Y^3+^- and Na^+^-ions and a slightly lower Na content, as described by Többens, then the conductivity could be lower [33]. In the case of the N5-Y composition, the effect of the N16.5 phase on the overall conductivity would be quite low as the content is lower than 5 vol.%. In contrast, the N5-Sm and N5-Gd contain considerable amounts of the respective N16.5-phases, therefore leading to a significant increase of the overall conductivity in this case. The ionic conductivities reported by Shannon and Okura are listed in Table 8 together with the reported crystallographic phases. In both cases, only the corresponding N5-type phases are described, and no uniform sequence of conductivities corresponding to the ionic radii of the rare earth elements used is given. However, both Sm-based compositions have higher conductivities than the Y-based composition. In the case of the Gd-based compositions, one sample has a higher conductivity than the Y-based sample and one sample has almost the same conductivity. In contrast, our data in Figure 8 attribute the highest conductivity to N5-Y, followed by N5-Gd and N5-Sm. This behavior has led us to assume that the N16.5 phases in N5-Gd and N5-Sm must have a considerably lower conductivity in comparison to the N5 phase, even if the ionic conductivity of this phase cannot be excluded. Additionally, Banks et al. showed temperature-dependent conductivity data for a set of glass-ceramic Na_5_RSi_4_O_12_ (R=Er, Y, Gd, Sm) compositions but reported no information of the phase content, therefore the data are not taken into account [21]. One must also be aware that the assumed tortuosity with a value of 1 brings some uncertainty, which varies with the content of both phases. However, the SEM images still do not allow a visual distinction between the N5 and the N16.5 phase, so quantifying this parameter from performed image analysis is impossible.

For activation energies, some discrepancy to earlier published work is found. The early investigations of Shannon et al. [19] have shown that Na^+^-ionic conductivity increases by substitution of Y by Gd and Gd by Sm in Na_5_RSi_2_O_12_ single crystals all demonstrating similar activation energies of 0.27…0.29 eV. This is also in agreement with the increasing lattice parameter and cell volumes of the phases (see Table 3). The activation energy of 0.14 eV for the Y-substituted composition must be rated as unusually low. Shannon at el. gives a value of 0.31 eV for hydrothermally synthesized Na_5_YSi_4_O_12_ and Yamashita et al. a value of 0.26 eV for an analogous composition prepared by spray drying and calcination [19,44]. Our investigations show a different trend for ionic conductivity regarding the size of the ionic radii. One possible explanation can be that conductivities, estimated in our work, contain grain and grain boundary contributions. The grain boundary contribution depends on the N16.5 phase and increases from the N5-Y to the N5-Sm sample. This can also explain the increase of activation energy and decrease of observed total conductivity because the grain boundaries not only have higher resistivity but also higher activation energy for ionic conductivity. Even if the indications for the contribution of both grain and grain boundary resistivities to the measured values of σ_N5_ in Figure 8 are strong, the performed measurements and investigations do not allow to make a decisive conclusion about the true reasons for the observed behavior and high-frequency measurements are necessary to clarify the open questions.

## 5. Conclusions

The sodium conducting glass-ceramic solid electrolyte having the general formula Na_5_RSi_4_O_12_ (R = Rare earth elements) is known for decades and only rarely investigated regarding a powder-based synthesis route which has several advantages in comparison manufacturing method from bulk glasses. In this work, sodium rare earth silicate glass frits NaRSiO containing the rare earth elements Y, Sm, and Gd according to the stoichiometric composition Na_5_RSi_4_O_12_ were prepared and processed into recrystallized powders. In contrast to the yttrium-based composition, denser microstructures can be achieved with substitution by samarium and gadolinium in analogous compositions at lower temperatures or shorter holding times. This behavior is primarily attributed to the effect of the larger ionic radii of these two elements on the sintering and melting temperatures, with larger ionic radii leading to lower temperatures. By XRD analysis, a mainly biphasic phase composition containing the conducting Na_5_RSi_4_O_12_ phase as the main component and a Na_16.5_(Na_0.5_R_0.5_)R_2_Si_12_O_36_ phase with a cubic crystal structure as an additional component was found. For both phases, increased ionic radii lead to a larger lattice dimension which is assumed to be the reason for the reduced melting and sintering temperatures. It is ambiguous whether the formation of the larger fractions of the Na_16.5_(Na_0.5_R_0.5_)R_2_Si_12_O_36_ phase in the case of the Sm- and Gd-based materials depend on the formation of rare-earth-rich phases or the sintering conditions. Using electrochemical impedance spectroscopy, the ionic conductivities of the N5 phases and the activation energy have been estimated. N5-Y phase has the highest conductivity with a value of 1.82 × 10^−3^ S cm^−1^ at 20 °C as well as the lowest activation energy. While the sintering characteristics of the individual compositions followed the decreasing melting temperatures with increasing ionic radii, an analogous behavior was not observed for the ionic conductivities. For a comprehensive overall understanding of the ionic conductivity of the multiphase microstructures, which also includes the grain boundaries and the minor phases, measurements at higher frequencies are required, which will be carried out in further work. In addition to the existing knowledge on the processing of yttrium-based, sodium-conducting rare earth silicates via the powder route, it has been demonstrated that this class of materials also shows a corresponding applicability using Sm and Gd. This opens more compositions of this class of solid electrolytes to the use of advanced shaping technologies such as screen printing and film casting, which are currently being discussed for solid-state battery manufacturing processes.

## Figures and Tables

**Table 1:** Conditions for crystallization of N5 phase from the corresponding parental glass compositions. Heating and cooling rate was 5 K/min.

Powder	Crystallization Temperature °C	Dwell Time h
N5-Y (Na_5_YSi_4_O_12_)	1100	1
N5-Gd (Na_5_GdSi_4_O_12_)	950	1
N5-Sm (Na_5_SmSi_4_O_12_)	950	1

**Table 2:** Conditions for sintering the pre-crystallized powders N5-Y, N5-Gd and N5-Sm. Heating and cooling rate was 5 K/min.

Powder	Sintering Temperature °C	Dwell Time h
N5-Y	1120	2
N5-Gd	1120	0.5
N5-Sm	1050	0.5

**Table 3:** Characteristic temperatures for sintering of pre-crystallized powders.

Powder Type	Shrinkage Start °C	Shrinkage End °C	Melting Point °C
N5-Y	961	1198	1213
N5-Gd	919	1134	1139
N5-Sm	865	1057	1082

**Table 4:** Pycnometric density of pre-crystallized powders and crystallographic density of corresponding Na_5_YSi_2_O_12_, Na_5_GdSi_2_O_12_ and Na_5_SmSi_2_O_12_ phase.

Powder Type	Pycnometric Density g/cm^3^	Crystallographic Density N5 g/cm^3^	Lattice Parameter of Na_5_RSi_4_O_12_, nm	
			a, nm	c, nm
N5-Y	2.84	2.87	2.2033	1.2618
N5-Gd	3.16	3.20	2.2156	1.2647
N5-Sm	3.12	3.14	2.2227	1.2661

**Table 5:** Results of indicative quantitative analysis of phase content of different crystalline phases in sintered N5-Y, N5-Gd and N5-Sm samples.

Sintered Sample	N5 Content wt.%	N16.5 Content wt.%	Lattice Parameter of Na_16.5_(Na_0.5_R_0.5_)R_2_Si_12_O_36_, nm	Crystallographic Density N16.5 g/cm^3^
N5-Y	95.8 ± 0.2	4.2 ± 0.2	1.5117	2.91
N5-Gd	72.6 ± 0.2	27.4 ± 0.2	1.5160	3.226
N5-Sm	65.9 ± 0,2	34.1 ± 0.2	1.5181	3.18

**Table 6:** Results of indicative quantitative analysis of the impedance spectra of N5-Gd sample measured at different temperatures including the calculation of relaxation frequency (Equation (2)) and apparent capacitance (Equation (3)).

T °C	R_G_ Ω cm	R_LF_ Ω cm	CPE_LF_	α_LF_	*f*_0,LF_ Hz	C_ap,LF_ F cm^−1^	Y_CPE_ S cm^−1^	α_CPE_
−10	1.50 × 10^4^	1.27 × 10^5^	1.43 × 10^−8^	0.87	231.08	5.43 × 10^−9^	7.89 × 10^−6^	0.94
0	1.02 × 10^4^	8.01 × 10^4^	1.44 × 10^−8^	0.87	369.93	5.37 × 10^−9^	1.25 × 10^−5^	0.95
10	6.30 × 10^3^	4.59 × 10^4^	1.51 × 10^−8^	0.88	646.35	5.36 × 10^−9^	2.18 × 10^−5^	0.94
20	4.52 × 10^3^	2.63 × 10^4^	1.22 × 10^−8^	0.90	1.19 × 10^3^	5.07 × 10^−9^	3.80 × 10^−5^	0.92
30	3.31 × 10^3^	1.62 × 10^4^	1.10 × 10^−8^	0.92	1.96 × 10^3^	5.02 × 10^−9^	6.20 × 10^−5^	0.90

**Table 7:** Results of indicative quantitative analysis of volumetric phase content of different crystalline phases in sintered N5-Y, N5-Gd and N5-Sm samples.

Sintered Sample	N5 Content $x_{N5}\;\mathrm{vol}.\%$	N16.5 Content $x_{N16.5}\;\mathrm{vol}.\%$	Porosity vol.%
N5-Y	89.5	3.9	5.99
N5-Gd	71.7	26.9	0
N5-Sm	66.1	33.8	0

**Table 8:** Literature of N5-type glass ceramics synthesized with the rare earth elements Y, Sm, and Gd according to Shannon et al. [19] and Okura et al. [34].

Nominal Composition	Phase	Temperature °C	Conductivity S cm^−1^	Source
Na_5_YSi_4_O_12_	N5	200	0.04	[19]
Na_5_GdSi_4_O_12_	N5	200	0.1	[19]
Na_5_SmSi_4_O_12_	N5	200	0.08	[19]
Na_3.9_Y_0.6_P_0,3_Si_2.7_O_9_	N5	300	0.066	[34]
Na_3.9_Gd_0.6_P_0,3_Si_2.7_O_9_	N5	300	0.063	[34]
Na_3.9_Sm_0.6_^P^_0.3_Si_2.7_O_9_	N5	300	0.13	[34]

## Data Availability

Not applicable.

## Supplementary Materials

The following are available online at https://www.mdpi.com/article/10.3390/ma15031104/s1, Figure S1: XRD pattern of sintered N5-Y sample in comparison with PDF cards of the N5 and N16.5 phases, Figure S2: XRD pattern of sintered N5-Gd sample in comparison with PDF cards of the N5 and N16.5 phases.
