# Supplementary material for: Influence of R=Y, Gd, Sm on Crystallization and Sodium Ion Conductivity of Na5RSi4O12 Phase
Source: Materials (Basel). 2022 Jan 30;15(3):1104. doi: 10.3390/ma15031104 (PMC8838226; doi:10.3390/ma15031104)
Supplement: Supplementary file 1 [file materials-15-01104-s001.zip › materials-1523499-supplementary.pdf]

materials

MDPI

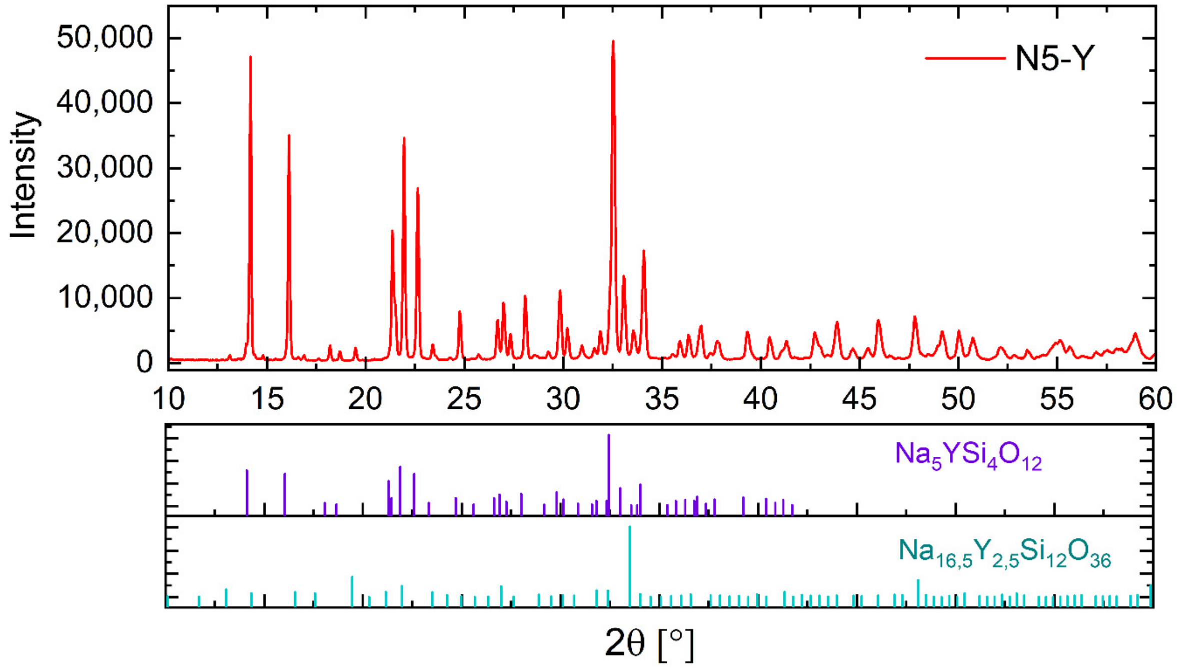

Figure S1: XRD pattern of sintered N5-Y sample in comparison with PDF cards of the N5 and N16.5 phases.

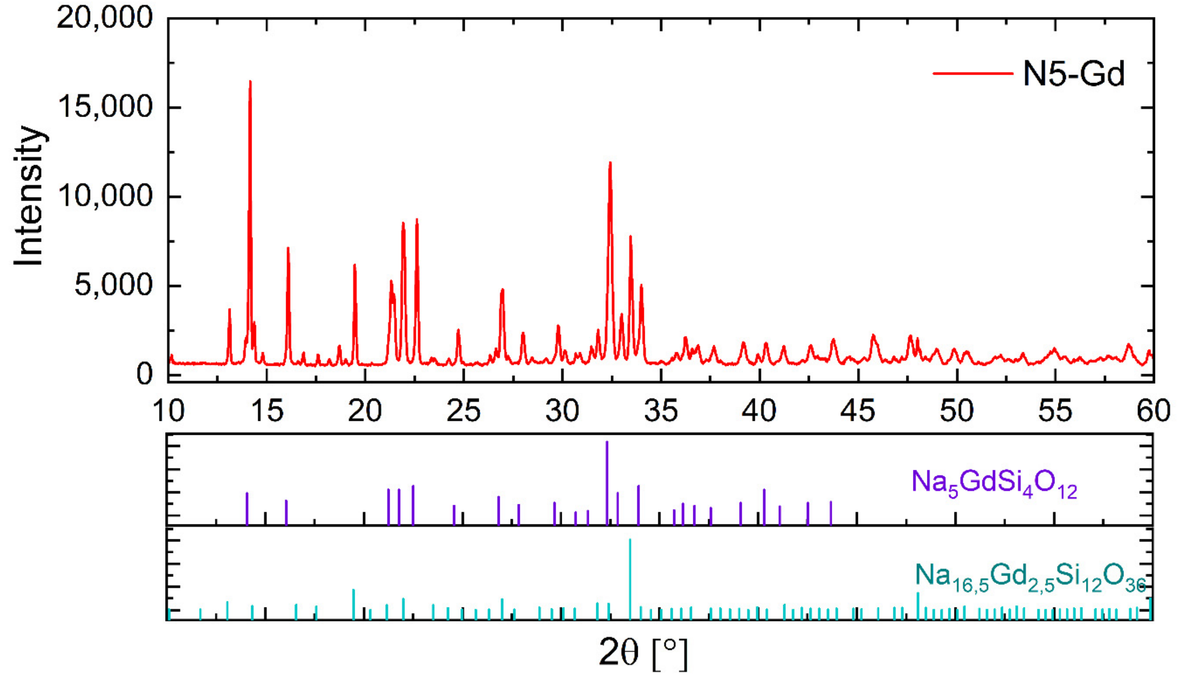

Figure S2: XRD pattern of sintered N5-Gd sample in comparison with PDF cards of the N5 and N16.5 phases
